# Neonatal mortality at the neonatal unit: the situation at a teaching hospital in Ghana

**DOI:** 10.4314/ahs.v18i2.22

**Published:** 2018-06

**Authors:** Benjamin Atta Owusu, Apiradee Lim, Nifatamah Makaje, Priscilla Wobil, Areeyuth SameAe

**Affiliations:** 1 Department of Mathematics and Computer Science, Faculty of Science and Technology, Prince of Songkla University, Pattani, Thailand; 2 Child Health Directorate, Komfo Anokye Teaching Hospital, Kumasi, Ghana

**Keywords:** Logistic regression, neonatal mortality, Kumasi

## Abstract

**Background:**

The first 28 days of life- the neonatal period is the most vulnerable time for a child's survival. Globally, neonatal mortality has seen a downward trend in recent years. The main objective of this study was to determine the percentage of neonatal mortality and to provide information on factors associated with neonatal mortality at the neonatal unit of a tertiary health facility or teaching hospital.

**Methods:**

Data of neonates admitted to the neonatal in-patient unit of the Komfo Anokye Teaching Hospital (KATH) in Ghana from January 2013 to May 2014 were analyzed. Logistic regression model was performed to assess the association between neonatal mortality and predictors.

**Results:**

A total of 5,195 neonatal admissions were recorded. The overall percentage of neonatal mortality was 20.2%. Infants with very low birth weight, having 5-minute Apgar score lower than 4, newborns with pre-term delivery, being referred from other health facilities, and being diagnosed with respiratory distress and birth asphyxia had a higher percentage of neonatal mortality.

**Conclusion:**

The mortality at the neonatal in-patient unit at the Komfo Anokye Teaching Hospital in Ghana is very high. There is the need for continuous attention and interventions to help reduce the risk of mortality among neonates admitted to the facility.

## Introduction

The first four weeks of life are the most crucial period of life. It is within this period that infants are highly prone to illness and death. Each year, almost 3 million newborns die within the neonatal period with half of these deaths occurring within the first 24 hours of birth^1^,^2^. Most of these deaths are attributable to infections, pre-term delivery and birth asphyxia^3^. The current pace of reduction in neonatal deaths could be accelerated through the provision of feeding support, extra care for pre-term newborns, improving resuscitation skills and infection prevention^4^. These solutions are achievable, however, neonatal mortality remains a big challenge for lower-middle income countries, especially in sub-Saharan Africa and Southern Asia with more than two-third of neonatal mortality occur in these regions^5^. Countries in these two regions have made the least progress towards reducing neonatal mortality^6^.

In Ghana, neonatal mortality rate was found at 39 per 1000 live births in 2012^19^ and decreased to 30 per 1000 live births in 2014^7^. However, neonatal mortality in Ghana remains higher than many other countries in Africa for example Ethiopia, Niger, Kenya, Malawi and Congo^8^. Thus, Ghana's Ministry of Health has launched the Ghana National Newborn Health Strategy and Action Plan 2014–2018 to reduce the neonatal mortality rate to 21 per 1000 live births^7^. Separate studies have identified place of birth, birth weight, mode of delivery, delayed breastfeeding initiation and age of baby at the time of admission as significant predictors of neonatal death^6^. Examining the risk factors of neonatal mortality at the various neonatal units is necessary as it allows inferences about the quality of care. It could provide insights into how neonates could be managed to improve the outcomes of admissions at the neonatal unit.

The neonatal unit at the Komfo Anokye Teaching Hospital (KATH), serves as the main referral destination for sick newborns from almost six out of the ten regions in Ghana. The unit is equipped with advanced life-saving equipment to support these sick newborns.

The aim of this study was to investigate the major causes and risk factors of neonatal mortality and estimate the percentage of neonatal mortality based on the number of newborns admitted to the neonatal unit, also called Mother and Baby Unit (MBU), KATH, Kumasi, Ghana.

## Methods

This was a hospital based study which used retrospective data from the neonatal in-patient unit to identify the major predictors of neonatal death at the hospital. This study was conducted at the MBU which is the neonatal in-patient unit of the KATH from January 2013 to May 2014. The KATH is a tertiary health facility located in a Kumasi, the capital of the Ashanti region and the most second most populous city in Ghana. KATH serves as the main referral facility for the Northern Sector of the country. The neonatal unit consists of three wards: a High Dependency Unit (HDU) that admits sick newborns referred from the delivery wards. There is also a pre-term or low birth weight (LBW) unit that admits pre-term or LBW newborns from the HDU who have been stabilized and a Septic unit that admits out-born newborns. Different categories of newborns are admitted to the facility. These include preterm newborns, low birth weight newborns, newborns with neonatal jaundice and sepsis and newborns with congenital anomalies. All neonates admitted to the unit from January 2013 to May 2014 were included in the study. These admissions included newborns who were delivered at different facilities and were referred to the unit on health grounds. The following information was retrieved from the in-patient treatment books: birth weight, sex, mode of delivery, place of delivery, age at admission, Apgar scores, time between birth and admission and outcome of admission. Information from 5,363 in-patient books was retrieved for the study. Out of this, 124 newborns were older than 28 days, hence were excluded from the study. Due to substantial missing information, 44 newborns were also excluded from the study. The newborns were grouped according to the place of delivery (whether they were delivered at KATH or referred from other facilities). Neonatal mortality was defined as death that occurred within the first 28 days of life.

The outcome of this study was neonatal mortality. It was defined as the number of neonates who died at the unit over the period of the study. This definition is found appropriate because all newborns at the neonatal unit are sick newborns brought in from either the hospital's delivery rooms or outside the hospital. Birth weights, mode of delivery, 5 minute Apgar score, gestational age, main diagnosis, gender and newborns' age at the time of admission were employed as exposure variables in this study. The birth weight was categorized into three groups namely; very low birth weight (VLBW), low birth weight (LBW), and normal weight. VLBW was defined as weight less than 1.5 kg; LBW for those between 1.5–2.4 kg, normal birth weight for those above 2.4 kg. The mode of delivery was divided into three categories. The first group is spontaneous vaginal delivery (SVD) which includes all neonates delivered by spontaneous delivery. The next category was vacuuming, made up all newborns delivered by vacuum extraction. Newborns delivered through caesarean section (C/S) were also categorized as a single group. Apgar score at 5 minutes was categorized in three groups; 0–3, 4–7 and above 7. Newborns' gestational age was classified as pre-term (< 36 weeks), term (> 36weeks) or not stated. The diagnosis was also classified into eight groups. The most prevalent conditions were prematurity, respiratory distress, infections, congenital anomalies, neonatal jaundice, sepsis and birth asphyxia. The age as of the time of admission was divided into four groups; 1 day, 2–7 days, 8–14 days and 15–28 days

### Statistical analysis

A preliminary statistical analysis was done to examine the frequency distribution of the variables and cross tabulation of the variables with outcome. Univariate analysis was done to examine the variables associated with neonatal death. These variables which were significant in the univariate analysis were consequently included in the logistic regression model. The logistic regression model was used to determine the strength of association between these predictors and the outcome while adjusting for confounders. Confidence interval graph based on sum contrast^9^ from the logistic model was constructed to illustrate the percent of neonatal deaths for each predictor. The normal quantile-quantile (Q-Q) plot was used to verify the normality assumption of the residuals from the model. The plot of observed count versus expected count of neonatal deaths was used to evaluate the fit of the model.

### Ethical approval

This study was approved by the Research and Development Unit (R&D) of KATH and the Committee on Human Research, Publications and Ethics, Kwame Nkrumah University of Science and Technology, School of Medical Sciences and KATH, Kumasi on with reference number CHRPE/AP/365/15.

## Results

Information on 5,195 newborns was used for the study. More than half of the newborns were males (55%). The percentage of neonatal mortality among males was 20% and that of females was 20.5% (Table 1). More than 80% (888) of neonatal deaths occurred in the first day of life with 17.1% mortality. Out of the 5,195 newborns admitted to the unit, 3,022 (58.2%) were delivered at the KATH while 2,173 (41.8%) were delivered and referred from outside the KATH. There were 1,053 neonatal deaths over the period. representing a percentage mortality of 20.3. Table 1 shows the distribution of the various variables and proportion of neonatal death.

**Table 1:** Distribution of neonatal characteristics

Variables	Survived (%)	Died (%)	p-value
***Gender***			0.7186
Female	1,860 (79.5)	480 (20.5)	
Male	2282 (80.0)	573 (20.0)	
***Birth Weight***			< 0.005
VLBW	431 (50.9)	416 (49.1)	
LBW	1,029 (85.3)	178 (14.7)	
Normal	2682 (85.4)	459 (14.6)	
***5 Minute Apgar score***			< 0.005
0–3	66 (32.7)	136 (67.3)	
4–7	644 (67.3)	313 (32.7)	
8–10	3,432 (85.0)	604 (15)	
***Gestational age***			< 0.005
Term	963 (91.7)	87 (8.3)	
Preterm	487 (68.5)	224 (31.5)	
Not Stated	2,692 (78.4)	742 (21.6)	
***Delivery Mode***			< 0.005
SVD	2,358 (76.2)	736 (23.8)	
C/S	1,736 (84.8)	311 (15.2)	
Vacuum	48 (88.9)	6 (11.1)	
***Place of Delivery***			<0.005
KATH	2,573 (85.1)	449 (14.9)	
Referred	1,569 (72.2)	604 (27.8)	
***Admission age***			< 0.005
1 day	3,210 (78.3)	888 (21.7)	
2–7 days	728 (85.1)	127 (14.9)	
8–14 days	102 (86.4)	16 (13.6)	
15–28 days	102 (82.3)	22 (17.7)	
***Diagnosis***			< 0.005
Prematurity	888 (69.1)	397 (30.9)	
Respiratory Distress	208 (72.2)	80 (27.8)	
Infections	1,120 (91.7)	102 (8.3)	
Congenital anomalies	377 (81.1)	88 (18.9)	
Neonatal Jaundice	799 (91.8)	71 (8.2)	
Birth asphyxia	638 (67.5)	307 (32.5)	
Others	112 ((93.3)	8 (6.7)	

From the univariate analysis there was no significant association of sex with neonatal mortality. Thus, it was excluded from the multivariate analysis. Other variables such as birth weight, 5-minute Apgar score, gestational age, delivery mode, admission age and main diagnosis were all significantly associated with neonatal mortality. From the logistic regression analysis, birth weight, place of delivery, 5-minute Apgar score, gestation and main diagnosis remained significantly associated with neonatal death. The logistic regression analysis also showed that the association of neonatal mortality with admission age and delivery mode disappeared. Compared to newborns born with Apgar score 8 or higher, newborns born with 5-minute Apgar score 4 – 7 and lower than 4 were more likely to have higher neonatal mortality of 2 and 8 folds, respectively. Very low birth weight newborns had higher mortality than normal weight newborns (7 times more). Newborns born pre-term had a 2 times increased in the risk of death higher than newborns with term born. Newborns referred to the neonatal unit had higher percent of mortality than newborns born at KATH. Crude and adjusted odds ratios (OR) for each variable are presented in Table 2.

**Table 2:** Crude and adjusted Odds ratio (OR) from the logistic regression analysis with 95% Confidence intervals

Variable	Crude OR (95%)	Adjusted OR (95% CI)	p-value
***Sex***			0.693
Female	1		
Male	0.97 (0.92, 1.03)		
***Birth Weight***			<0.005
Normal	1	1	
LBW	1.01 (0.84, 1.22)	1.23 (0.98, 1.55)	
VLBW	5.64 (4.77, 6.66)	7.24 (5.40, 9.69)	
***5 Minute Apgar score***			<0.05
8–10	1	1	
4–7	2.76 (2.35, 3.24)	2.06 (1.69, 2.50)	
0–3	11.71 (8.62, 15.91)	8.27 (5.81, 11.79)	
***Gestational age***			<0.05
Term	1	1	
Preterm	5.09 (3.88, 6.67)	2.23 (1.60, 3.12)	
Not Stated	3.05 (2.41, 3.86)	1.96 (1.50, 2.55)	
***Delivery Mode***			0.553
C/S	1		
SVD	1.74 (1.51, 2.01)		
Vacuum	0.70 (0.30, 1.64)		
***Place of Delivery***			<0.05
KATH	1	1	
Referred	2.21 (1.92, 2.53)	2.40 (2.00, 2.87)	
***Admission age***			0.444
1 day	1		
2–7 days	0.63(0.51, 0.77)		
8–14 days	0.57 (0.33, 0.97)		
15–28 days	0.78 (0.49, 1.24)		
***Diagnosis***			<0.05
Others	1	1	
Prematurity	6.26 (3.03, 12.95)	1.02 (0.47, 2.13)	
Respiratory distress	5.38 (2.51, 11.44)	4.00 (1.82, 8.79)	
Infections	1.28 (0.61, 2.89)	0.84 (0.39, 1.81)	
Congenital anomalies	3.27 (1.54, 6.94)	2.11 (0.97, 4.57)	
Neonatal Jaundice	1.24 (0.58, 2.65)	0.70 (0.32, 1.54)	
Birth asphyxia	6.74 (3.25, 13.98)	3.17 (1.49, 6.74)	

**Figure 1:**
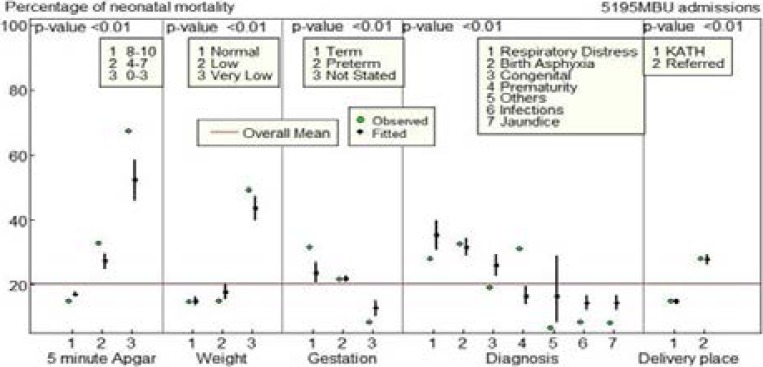
Mortality rate of each variable used in the logistic regression model

The observed and adjusted proportions of variables that were significant from the multivariate analysis are presented in Figure 1 together with the overall mean percentage mortality. Newborns with birth asphyxia, congenital anomalies, infections and respiratory distress had higher neonatal mortality than the overall mean. The horizontal line indicates the overall mean.

**Figure 2:**
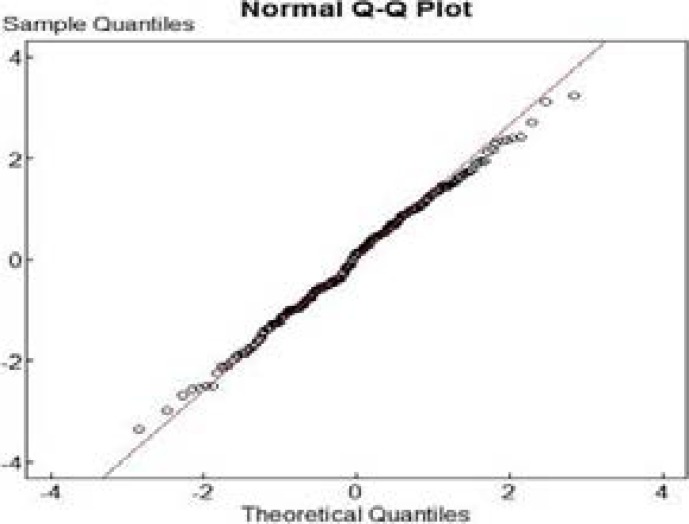
Normal quantile-quantile plot of residuals

**Figure 3:**
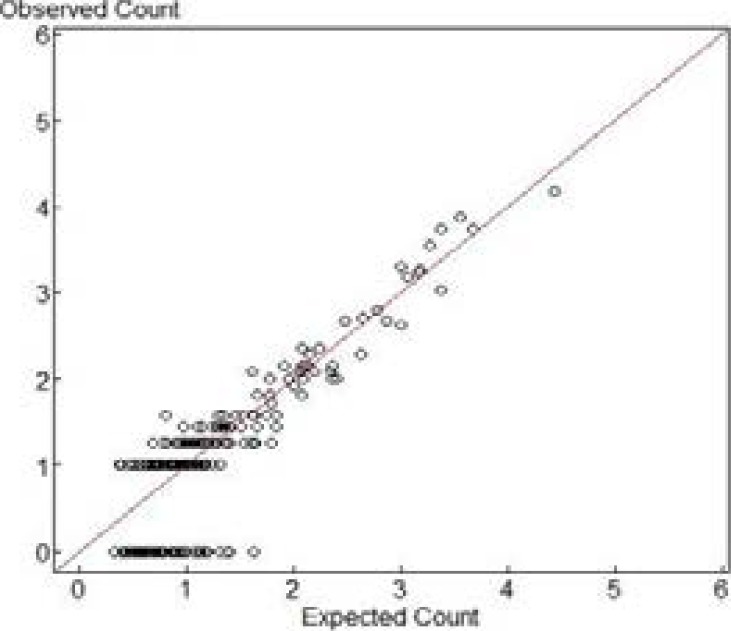
Expected and observed values

Quantile-Quantile (Q-Q) plot (Figure 2) of the residuals from the logistic regression model indicates that the model has provided an acceptable fit for the outcome variable (neonatal mortality). It also shows that the estimates from the model are asymptotically normal. A plot of the observed and fitted values is also provided in figure 3. The values match each other in a vertical pattern.

## Discussion

Our study revealed that the survival of a neonate in the neonatal unit depended on the birth weight, 5-minute Apgar score, place of delivery, gestational age and discharge diagnosis. Other population and hospital based studies conducted in Pakistan and the Korle-Bu Teaching Hospital in Ghana have found these factors to be associated with neonatal death^10^,^11^. Although different researches have documented evidence to show higher mortality percentages in male than female infants^12^,^13^, our study found otherwise. We found no significant difference in the mortality of males and females. However, the reasons for such contrast result could not be explained from the result. The percentage of neonatal death for newborns delivered at KATH was 15% while that of newborns referred from other health facilities was 27.6% The overall mortality at the neonatal unit was 20.3%. This result is lower than 29.1% and 22.4% that was recorded for the neonatal intensive care unit of the Cairo university children's hospital, Egypt and Abha Hospital, Saudi Arabia^13^,^14^. However, percentage of mortality among newborns referred to the facility still remains very high. This is because most newborns referred to KATH are usually very sick and require specialist care that is not provided by primary health facilities.

Although gestational age was a significant risk factor, it was only significant among newborns delivered at KATH. Pre-term birth was associated with a higher risk of death and higher mortality percentage more than the overall percentage. This is comparable with results presented by Lawn^3^ and Hsu^6^. Pre-term newborns require special care from skilled workers to improve their chances of survival. Such special care involves providing warmth and feeding support^4^. The major conditions associated with neonatal death were infections, respiratory distress, prematurity, neonatal jaundice, sepsis, infections birth asphyxia and other neonatal conditions. Recent population and hospital based studies have also corroborated these findings^10^,^15^,^16^. Among these neonatal conditions, birth asphyxia recorded the highest death rate. Clean birth practices have shown to significantly reduce neonatal mortality. Basic well known hygienic practices such as hand washing and maintaining a clean environment are poorly observed^4^. In this present study birth weight was a major risk factor of neonatal death. The results revealed that VLBW had higher risk of neonatal mortality than newborns with normal weight. Other studies in sub-Saharan Africa^17^,^18^ and Asia^14^ have made similar findings to the effect that birth weight is a significant risk factor of neonatal mortality. However, the odds ratio of death for low birth weight infants is lower than what was reported in a five-year study conducted at the facility in 2012^19^. This shows that the MBU of the KATH has made great strides in saving low birth weight infants.

### Study limitation

This was a hospital based study and not a representation of the entire population in the city. Although the annual deliveries figure for the hospital exceed 10,000, this study focused only on sick neonates. Data for this study is retrospective and thus, there were cases of missing information although not significant.

## Conclusion

The neonatal mortality percentage at the neonatal unit of the KATH is still high. This study found that newborns referred from home or other health facilities, newborns having very low birth weight, 5 minute Apgar score of less than 4, preterm birth and having congenital anomalies were more likely to have a higher percentage. There is, therefore, the need for continuous attention and strengthening of new-born interventions to help reduce the risk reduce the risk of mortality among neonates delivered at KATH
